# Monitoring of mental health in occupational populations: a study on the role and application of HDL-related inflammatory index

**DOI:** 10.3389/fpubh.2025.1563742

**Published:** 2025-03-31

**Authors:** Ma Jingxuan, Pingcuo Yuzhen, Li Zhen, Wang Juan, Wang Hongjian, Lan Yajia

**Affiliations:** ^1^West China School of Public Health, Sichuan University, Chengdu, China; ^2^Chengdu Disease Prevention and Control Center, China Railway Chengdu Bureau Group Co., Ltd., Chengdu, China

**Keywords:** occupational monitor, HDL, inflammatory index, stress, depression, anxiety

## Abstract

**Background:**

Mental disorders in occupational populations pose significant health and economic burdens, but there exists a lack of practical and objective biomarkers for occupational mental health monitoring. Our study aims to explore the correlation between high-density lipoprotein (HDL)—related inflammatory markers and negative psychological symptoms in occupational populations. We also seek to evaluate the potential application effectiveness of these indicators as biomarkers for identifying the impact of mental health on occupational populations. Moreover, the indicators found in this study can be used as indicators for identifying high-risk groups prone to inflammatory responses caused by negative psychological symptoms.

**Methods:**

Our study adopted a cross-sectional design with a combination of questionnaires and biochemical index tests for 1920 eligible occupational populations. The Depression Anxiety Stress Scales-21 (DASS-21) was used to measure participants’ levels of anxiety, depression, and stress. Collect individual and occupational characteristics of survey respondents through self-administered questionnaires. Blood samples are also collected to measure high-density lipoprotein cholesterol levels and peripheral blood cell counts. We employed statistical analyses including correlation analysis, Receiver Operating Characteristic (ROC) curve analysis, and univariate and multivariate regression.

**Results:**

The final sample size included in the analysis was 1,434. The results showed that stress, anxiety, and depressive symptoms were significantly correlated with all four HDL-related inflammatory indices (*p* < 0.05). Especially for MHR, compared to those without symptoms, individuals experiencing stress, anxiety, or depression had an OR of 2.75 (95% CI: 1.90, 3.99), 3.27 (95% CI: 2.25, 4.78), and 3.02 (95% CI: 2.08, 4.40) for abnormally high levels, respectively. In addition, subgroup analyses showed that lower monthly incomes, longer working hours and frequent night shifts might be promoting factors for elevated HDL-related inflammatory levels. Receiver Operating Characteristic (ROC) analysis further demonstrated that PHR and MHR exhibit good predictive ability for all three psychological symptoms, with AUC values exceeding 0.6. Notably, for individuals with over 30 years of work experience, the predictive performance AUC is even higher, reaching above 0.7.

**Conclusion:**

The results of this study suggest that PHR and MHR are expected to be potential biomarkers for identifying health problems caused by negative psychological symptoms in occupational groups, providing valuable information for occupational mental health assessment. Preventive measures should be implemented for high-risk groups, including those with low income, long working hours, and frequent night shifts, to mitigate potential health impacts.

## Introduction

Mental health is the cornerstone of overall well-being, exerting a significant impact on both individual health and the stability of societal systems. In 2019, the World Health Organization (WHO) reported that approximately 97 million individuals, nearly one-eighth of the global population suffered from mental disorders. Among these disorders, anxiety and depression were identified as the most common types (1). The outbreak of COVID-19 in 2020 led to a substantial surge in the incidence of anxiety and depressive disorders, with preliminary data indicating that within just 1 year, the prevalence of anxiety disorders and major depressive disorders increased by 26 and 28%, respectively (2). In China, the incidence rates of major depressive disorder and anxiety disorders in 2019 were 3519.26 and 3363.60 per 100,000 people, respectively. Depression, anxiety are leading contributors to disability-adjusted life years (DALYs), accounting for 37.26 and 22.54% of the total DALYs attributed to mental disorders in the Chinese population in 2019 (3). The China National Mental Health Development Report (2021–2022) found that the detection rates of depression and anxiety risks among adults were 10.6 and 15.8%, respectively (4). Occupation is a significant factor affecting mental health, with job burnout having a pronounced impact on the risks of depression and anxiety (5), with the detection rate of depression risk exceeding 40% in groups experiencing severe job burnout (6). Mental health issues among occupational populations can lead to sleep disorders and cardiovascular diseases (7, 8). Moreover, they can result in more severe consequences for employee physical and mental health, such as overwork death and suicide (9, 10). The societal impact of workers’ mental health extends beyond individual well - being. Psychological challenges among employees can lead to workplace accidents, increased absenteeism, and higher medical and insurance costs. These issues not only affect the workers themselves but also result in significant economic losses for businesses and the broader economy due to reduced productivity (11–13).

Identifying and applying effective monitoring indicators is crucial for surveillance of employees’ mental well-being, enhancing work efficiency, and promoting career development. According to the past experience, the predominant tools are various psychometric scales (14–16). These scales predominantly rely on the subjective experiences of participants, which can be swayed by transient emotional inclinations. Furthermore, these scales may not effectively gage the extent of the physiological emotional load that the body is experiencing. There is a realistic need to identify biomarkers that directly correlate psychological symptoms with physical health outcomes. Such biomarkers would offer a more direct and objective view of the potential health risks lurking beneath the surface and provide a clearer understanding of the health impairments caused by negative emotions. By clarifying these connections, we can gain a more refined understanding of the physical consequences of psychological distress, thereby enhancing the precision and effectiveness of health management strategies for working populations.

Research has pinpointed biomarkers associated with negative psychological symptoms, including cortisol, adrenaline, DHEA, and 8-OHdG (17–20). These indicators exhibit a moderate correlation with real-time psychological fluctuations; however, they are inadequate in capturing the cumulative effects of chronic emotional stress (21–24). The majority of these biomarkers necessitate complex and costly laboratory techniques, and their measurement may be susceptible to diurnal variations (25–28). Consequently, there is an urgent need to identify more precise and stable biomarkers, particularly those capable of capturing the enduring impacts of psychological status.

As a common surveillance item in occupational health checkup, high-density lipoprotein cholesterol (HDL-C) can serve as an effective indicator for monitoring health damage (29). Usually, HDL-C is recognized as beneficial “good cholesterol” for health (30). The level of HDL-C not only serves as an indicator of the body’s capacity to regulate cholesterol metabolism but is also intricately linked to the risk evaluation for a spectrum of diseases (31, 32). HDL-C levels are influenced by a multitude of factors, including genetics, lifestyle choices, illness, and pharmacological treatments (33–36). Additionally, they are closely tied to psychological factors and conditions, highlighting the complex interplay between physical and mental health. Occupationally induced negative psychological states may precipitate alterations in HDL-C levels. Luciano’s investigation revealed that healthcare workers subjected to occupational stress exhibit diminished concentrations of HDL-C, correspond with those from comparable studies in other occupational populations (37–41). Although there is a link between HDL-C and poor psychological status, current research does not endorse HDL-C as a reliable biomarker for overall health issues. Its accuracy is inadequate, as it mainly reflects lipid metabolism problems rather than offering a comprehensive view of systemic health (42–44). No solitary biomarker possesses the capacity to encapsulate the entirety of health dimensions. Consequently, while high-density HDL-C plays a critical role in lipid metabolism, it remains inadequate for a thorough health evaluation (45–47). The adoption of more extensive and integrated biomarker panels is imperative for a holistic assessment of health.

Recently, the lymphocyte to HDL-C ratio (LHR), monocyte to HDL-C ratio (MHR), neutrophil to HDL-C ratio (NHR), and platelet to HDL-C ratio (PHR) have emerged as novel biomarkers (48, 49). These biomarkers are defined as the ratios of specific blood cell counts, including lymphocytes, monocytes, neutrophils, and platelets, to the levels of HDL-C. The potential mechanism of inflammatory indices such as MHR, LHR, NHR, and PHR lies in their ability to reflect the activity and proportions of immune cells and their distribution changes relative to high-density lipoprotein in response to external stress. These biomarkers may yield valuable insights for the evaluation of various diseases, including hypertension, stroke, cardiovascular disease (50–52), periodontitis and prediction of affective disorders such as bipolar disorder, schizophrenia, and depression (53–56). However, the application value of these indicators in the healthy population, especially in the occupational population, has not been sufficiently studied and explored, especially in the field of occupational psychology. HDL related inflammatory indicators often showed different performance in different populations, which may be related to age, income, gender, and work factors. For example, CHEN’s research shows that LHR is a risk factor for depression in people over 60 years old, and family economic level is also an important stratification factor; Qing et al. also indicated that individuals over the age of 50, females, low-income individuals, and those with low education levels seem to have HDL related indicators as risk factors for depression (57, 58). As a comprehensive reflection of economic and educational levels, it is necessary to divide and analyze occupations into subgroups.

The study tries to explore a more convenient and economical detection method to gain insights into the inflammatory condition and psychological health levels within the occupational population. Integrating biological indicators with psychological scales allows us to capture not only the profound psychological distress that individuals subjectively report but also the underlying physiological reactions triggered by negative psychological symptoms. This composite assessment strategy aids in comprehensively understanding the potential health impact of workplace psychological status and facilitates timely identification of high-risk groups by employers for precision health management. Additionally, this research provides empirical data support for policymakers in developing monitoring and intervention strategies for occupational mental health.

## Method

### Study subjects

We selected three types of labor industries and used cluster sampling to collect samples from units that participated in routine medical examinations at a tertiary hospital within a specified time frame according to inclusion and exclusion criteria. We selected the subjects for cluster sampling based on the workplace characteristics, data accessibility, and the participants’ cooperation. The final sample consisted of employees from three specific work establishments: an educational institution, a healthcare facility, and an architectural design company. All eligible individuals, as determined by the inclusion–exclusion criteria, were included in the study. The eligibility criteria for participation in this survey were as follows: (1) voluntary consent to participate; (2) status as a regular employee for a minimum of 1 year; (3) absence of mental illness or cognitive deficits; (4) overall health with no history of chronic metabolic diseases and acute infectious diseases. Exclusion criteria included: (1) submission of questionnaires with inadequate quality; (2) retirement from employment; (3) acute or chronic infection occurred within the past month; (4) diagnosis of hyperlipidemia, hypertension, hyperglycemia, or other metabolic disorders; (5) incomplete physical examination records. A total of 1,920 employees meeting the research criteria were enrolled in the study. All participants provided informed consent for the questionnaire content and completed the survey anonymously. The investigation was conducted in adherence to medical ethical standards. This study has been approved by the Ethics Committee of West China School of Public Health, Sichuan University (Gwll2024120).

**Figure 1:**
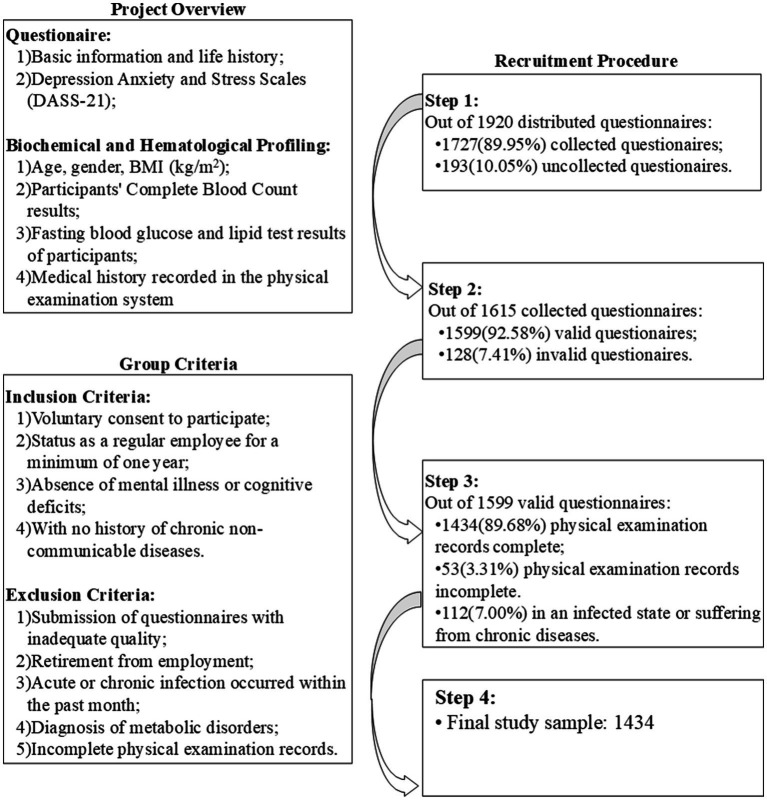
The main research process of this study.

The survey was carried out by a team of professionals with expertise in occupational health and epidemiology. The study coordinator delineated the objectives of the research and highlighted key considerations for the survey. Subsequently, the investigators were grouped and tasked with explaining the purpose and significance of the survey to the on-site participants, as well as guiding them through the questionnaire completion process. The survey content primarily encompassed demographic characteristics (age, gender, height, weight, educational level, marital status, lifestyle, etc.), occupational attributes (occupation, type of work, position, working hours, etc.), and physical and mental health status. A total of 1,920 questionnaires were distributed, with a return of 1,615, of which 1,599 were deemed valid, resulting in a response rate of 89.68%, whose effective rate of 92.58%. In this study, we integrated self-reported questionnaire data with existing physical examination records. Due to the occurrence of non-participation in the health check-up among the participants, the final sample size comprised 1,434 individuals for the subsequent analytical procedures, the specific research process is shown in Figure 1.

### Questionnaire

#### Basic information and life history

A self-designed demographic survey questionnaire was employed to collect data on demographic characteristics, encompassing gender, age, educational level, and income. Educational level was categorized according to academic degree, with divisions into specialized training and below, bachelor’s degree, and postgraduate and above. Income levels were stratified as follows: below 3,000 yuan, 3,001 ~ 5,000 yuan, 5,001 ~ 10,000 yuan, and above 10,001 yuan. Individual occupational characteristics included working hours, position, type of work, years of service, and frequency of night shifts. In this study, working hours were defined as the number of hours worked per week and were categorized into ≤40 h/week, 41 ~ 48 h/week, 49 ~ 55 h/week, and > 55 h/week. Position referred to the current job role of individuals with more than 1 year of work experience and included workers, technician, teachers, administrators, medical staff. Work years were divided into 1 ~ 10 years, 11 ~ 20 years, 21 ~ 30 years, and above 30 years. Night shift frequency was categorized as <1 times/week, 1 ~ 2 times/week, 3 ~ 4 times/week, and ≥5 times/week. Lifestyle history included the frequency of alcohol consumption, smoking, and physical exercise. Drinking was classified as non-drinker (never consumed alcohol), less than once a week, 1 ~ 4 times/week, more than five times a week; smoking was categorized as non-smoker (never smoked), sometimes smoker (less than once a week), usually smoker (more than once a week) and those who quit smoking; exercise frequency was divided into 0 times/week, 1 ~ 2 times/week, 3 ~ 4 times/week, and ≥ 5 times/week.

#### DASS-21 scale

A translated and revised version of the abbreviated form of the Depression Anxiety and Stress Scales (DASS-21) is utilized to measure negative psychological symptoms. This scale comprises 21 items, which are divided into three dimensions: anxiety, depression, and tension, each consisting of seven items. Each item is rated on a 4-point scale: 0 = does not apply to me at all, 1 = applies to me sometimes, 2 = applies to me often, 3 = applies to me very often (59). The scores for each dimension are multiplied by 2 to obtain the total score for that dimension, with a possible range of 0 to 42. Higher scores indicate a more severe degree of anxiety, depression, or tension. In order to observe the physiological changes caused by negative psychological symptoms, the scores of each subscale of DASS-21 were divided into binary variables based on their distribution, and the cut-off value was determined to have the symptom according to statistical conventions, based on the 75th percentile. The criteria for the three dimensions are as follows: a score of ≥14 on the anxiety dimension, ≥14 on the depression dimension, and ≥18 on the tension dimension is considered indicative of the presence of corresponding occupational negative affect. The DASS-21 has been widely employed to assess negative affect in various populations and has demonstrated good reliability and validity. In the present study (60, 61), the Cronbach’s alpha coefficients for the full scale, as well as the anxiety, depression, and tension subscales, were 0.94, 0.82, 0.87, and 0.86, respectively.

#### Biochemical and hematological profiling

Patient code, encompassing age, sex, diagnostic status, body mass index (BMI), and laboratory results, were meticulously documented. BMI classifications were based on the standards set forth in the Dietary Guidelines for Chinese Residents (2022), where in a BMI range of 18.5 kg/m^2^ to 23.9 kg/m^2^ was considered indicative of a healthy adult weight, 24 kg/m^2^ to 27.9 kg/m^2^ indicated overweight, and BMI of 28 kg/m^2^ or higher was classified as obese (62). Venous blood samples were procured from all participants on the day of questionnaire administration, specifically during the early morning hours. These samples were processed by laboratory staff who were blinded to the patients’ clinical diagnoses. Complete anticoagulation of the blood samples was achieved using EDTA-K2, and analyses were conducted within a 2-h window post-collection. Hematological indices, including erythrocyte count, hemoglobin concentration, leukocyte count, and differential leukocyte count (comprising neutrophils, lymphocytes, monocytes, eosinophils, and basophils), were determined utilizing an automated hematology analyzer in conjunction with corresponding reagents. Serum cholesterol and HDL-C levels were measured using an automated biochemical analyzer with appropriate reagents. Novel hematology-based ratios, including the Neutrophil-to-HDL Ratio (NHR), Monocyte-to-HDL Ratio (MHR), Lymphocyte-to-HDL Ratio (LHR), Platelet-to-HDL Ratio (PHR) were computed using the following equations: NHR = neutrophil count/HDL level; MHR = monocyte count/HDL level; LHR = lymphocyte count/HDL level; PHR = platelet count/HDL level.

#### Statistical analysis

Data verification and validation were performed by two experienced investigators, followed by data entry and organization using Microsoft Excel 2021 software. Quantitative variables were characterized by their mean ± standard deviation, whereas qualitative variables were detailed through frequency and proportion. In the absence of a recognized medical reference range for HDL related indicators, this study defines a value distribution of over 90% of the indicator as abnormally high. Chi-square tests were utilized to compare the presence of differences in anxiety, depression, and stress across various characteristics. Student’s t-tests were applied to describe and compare differences in inflammatory markers based on hematological cytology examination between occupational groups with and without stress, anxiety and depression. Multivariate logistic regression analysis is adopted, incorporating demographic information as well as stress, anxiety, and depression status, to observe whether there is an abnormal increase in HDL-related indices under different negative psychological states. Perform subgroup analyses to identify the sensitive occupational groups. Evaluate the predictive performance of the identified indicators using the area under the curve (AUC), accuracy (ACC), sensitivity (SEN), specificity (SPE), and Youden’s index. Statistical analysis of the data was performed using R version 4.2.1, with core packages including ReportROC, jstable, and tidyverse (63–65).

## Results

### Demographic characteristics and associations with psychological symptoms

Table 1 elucidates the statistically significant associations between a spectrum of demographic and lifestyle variables and the prevalence of stress, anxiety, and depression. Gender emerges as a pivotal determinant across all three mental health outcomes, with males demonstrating elevated rates of stress, anxiety, and depression (*p* < 0.05) relative to females. Age group significantly correlates with these symptoms, particularly anxiety and stress, where individuals aged 40 and over report increased prevalence (*p* < 0.001). Marital status is notably associated with stress, anxiety, and depression (*p* < 0.001), indicating that divorced or widowed individuals are at an elevated risk. Educational attainment exhibits a robust correlation with stress, anxiety, and depression (*p* < 0.001), suggesting that individuals with lower levels of education are more prone to these symptoms. Income level is significantly associated with all three symptoms, with the lowest income bracket (≤3,000 yuan per year) exhibiting the highest prevalence of stress, anxiety, and depression (*p* < 0.001).

**Table 1:** Comparation of emotional symptom rates by demographic and occupational characteristics.

Features	*N*	Stress			Anxiety			Depress		
		Case	Rate (%)	*p*	Case	Rate (%)	*p*	Case	Rate (%)	*p*
**Sex**				0.009			<0.001			0.002
Male	645	194	30.1		227	35.2		225	34.9	
Female	789	188	23.8		192	24.3		214	27.1	
**Age (year)**				0.032			<0.001			0.022
20–	465	114	24.5		134	28.8		131	28.2	
30–	567	139	24.5		131	23.1		160	28.2	
40–	269	84	31.2		102	37.9		103	38.3	
≥50	133	45	33.8		52	39.1		45	33.8	
**Marriage**				0.003			<0.001			<0.001
Married	1,009	276	27.4		307	30.4		315	31.2	
Divorced or widowed	50	22	44.0		26	52.0		26	52.0	
Unmarried	375	84	22.4		86	22.9		98	26.1	
**Education**				<0.001			<0.001			<0.001
College degree or below	205	69	33.7		95	46.3		86	42.0	
Bachelor degree	760	240	31.6		262	34.5		269	35.4	
Graduate degree or above	469	73	15.6		62	13.2		84	17.9	
**Monthly income (yuan)**				<0.001			<0.001			<0.001
≤3,000	73	34	46.6		37	50.7		33	45.2	
3,001–	346	122	35.3		157	45.4		155	44.8	
5,001–	499	129	25.9		135	27.1		148	29.7	
≥10,000	516	97	18.8		90	17.4		103	20.0	
**BMI**				0.192			<0.001			0.009
Slim	136	45	33.1		59	43.4		46	33.8	
Normal	736	188	25.5		205	27.8		211	28.7	
Overweight	438	111	25.3		111	25.3		126	28.8	
Fat	124	38	30.6		44	35.4		46	37.1	
**Exercise (times/Week)**				0.082			0.772			0.067
<1	512	154	30.1		155	30.3		176	34.4	
1 ~ 4	808	198	24.5		230	28.5		232	28.7	
≥5	114	30	26.3		34	29.8		31	27.2	
**Smoke**				0.544			0.18			0.298
Never	1,155	300	26		333	28.8		347	30.0	
Sometimes	82	26	31.7		28	34.1		29	35.4	
Usually	152	45	29.6		50	32.9		53	34.9	
Quit smoking	45	11	24.4		8	17.8		10	22.2	
**Drink (times/Week)**				<0.001			<0.001			<0.001
Never	606	177	29.2		189	31.2		168	27.7	
<1	564	94	16.7		98	17.4		113	20	
1 ~ 4	220	95	43.2		107	48.6		104	47.3	
≥5	44	16	36.4		25	56.8		20	45.5	
**Night work (times/week)**				0.008			<0.001			<0.001
<1	656	147	22.4		155	30.2		168	32.7	
1 ~ 2	409	129	31.5		156	55.3		155	55.0	
3 ~ 4	234	68	29.1		73	44		77	46.4	
≥5	135	38	28.1		35	34		39	37.9	
**Work Type**				<0.001			<0.001			<0.001
Workers	109	27	24.8		39	35.8		36	33.0	
Technician	713	128	18		115	16.1		141	19.8	
Teachers	78	8	10.3		14	17.9		15	19.2	
Administrators	183	44	24		44	24		43	23.5	
Medical staff	351	175	49.9		207	59		204	58.1	
**Work Years**				0.010			<0.001			0.001
1~	753	185	24.6		194	25.8		213	28.3	
11~	385	96	24.9		102	26.5		108	28.1	
21~	182	59	32.4		72	39.6		75	41.2	
≥30	114	42	36.8		51	44.7		43	37.7	
**Weekly work hours**				<0.001			<0.001			<0.001
≤40	262	79	30.2		98	37.4		97	37.0	
41~	427	67	15.7		136	31.9		73	17.1	
49~	403	107	26.6		71	17.6		125	31.0	
57~	342	129	37.7		114	33.3		144	42.1	

Body mass index (BMI) category is statistically significant for anxiety (*p* < 0.001) and depression (*p* = 0.009), with overweight and obese individuals experiencing a higher prevalence. Smoking status does not achieve statistical significance for any of the symptoms. However, alcohol consumption is significantly associated with all three, with the highest rates observed among those who consume alcohol more frequently (*p* < 0.001). Frequency of night work is significantly associated with stress, anxiety, and depression (*p* < 0.05), highlighting frequent night work as a risk factor. Occupation type also emerges as a significant factor, with medical staff exhibiting the highest rates of stress, anxiety, and depression (*p* < 0.001). Both work experience and weekly working hours are significant predictors, with longer work years and increased work hours per week linked to higher rates of stress, anxiety, and depression.

### HDL-related inflammatory indices and psychological symptom status

Table 2 shows the distribution of blood cells, blood glucose, and blood lipids in the study subjects. The results show that in the physical examination hematological indicators of the study samples, except for hemoglobin and phagocytic granulocytes, all other indicators show a skewed distribution, with a left skewed peak.

**Table 2:** The distribution of blood cells, blood glucose, and blood lipids of the samples.

Index	Min-Max	Median ± IQR	Mean ± Sd
TC (mmol/L)	2.16–8.72	4.42 ± 1.05	4.53 ± 0.85
LDL-C (mmol/L)	2.84–7.37	2.84 ± 1.04	2.90 ± 0.79
HDL-C (mmol/L)	1.26–2.85	1.26 ± 0.38	1.30 ± 0.31
RBC (×10^12^/L)	4.83–7.05	4.83 ± 0.68	4.86 ± 0.51
WBC (×10^9^/L)	5.85–14.02	5.85 ± 1.78	6.03 ± 1.45
LC (×10^9^/L)	1.95–4.96	1.95 ± 0.68	1.98 ± 0.53
NC (×10^9^/L)	3.35–9.70	3.35 ± 1.35	3.52 ± 1.08
MONO (×10^9^/L)	0.34–0.99	0.34 ± 0.15	0.36 ± 0.12
PLT (×10^9^/L)	223.00–435.00	223.00 ± 63.75	229.21 ± 49.90
EOS (×10^9^/L)	0.11–1.05	0.11 ± 0.10	0.14 ± 0.11
BAS (×10^9^/L)	0.03–0.11	0.03 ± 0.02	0.03 ± 0.02
Hb (g/L)	146.00–187.00	146.00 ± 20.00	144.49 ± 14.44
GLU (mmol/L)	4.91–20.45	4.91 ± 0.56	5.02 ± 0.79
PHR	177.46–673.33	177.46 ± 73.00	185.90 ± 62.62
NHR	2.70–11.31	2.70 ± 1.48	2.88 ± 1.21
MHR	0.27–1.33	0.27 ± 0.15	0.30 ± 0.14
LHR	1.51–6.87	1.51 ± 0.80	1.62 ± 0.65

TC, Total cholesterol; LDL, Low density lipoprotein cholesterol; HDL, High-density lipoprotein cholesterol; RBC, Red blood cell count; WBC, White blood cell count; LC, Lymphocyte cell; NC, Neutrophils cell; Hb, Hemoglobin; MONO, Mononuclear cell count; PLT, Platelet; EOS, Eosinophils count; BAS, Basophil granulocyte count: GLU, Blood glucose; PHR, Platelet-to-HDL ratio; NHR, Neutrophil-to-HDL ratio; MHR, Monocyte-to-HDL ratio; LHR, Lymphocyte-to-HDL ratio.

Supplementary Table S1 provides an in-depth analysis comparing four key indices for individuals under normal conditions versus those experiencing stress, anxiety, or depression. Among the stress group, all indices were markedly higher than the normal group, with statistical significance (*p* < 0.05). The findings encapsulated in Supplementary Table S1 underscore the presence of pronounced differences in these four indices among individuals grappling with stress, anxiety, and depression, as opposed to those exhibiting normal mental health parameters.

### Associations between HDL-related inflammatory indices and negative psychological symptoms

For the relationship between psychological symptoms and HDL related inflammatory indicators, the correlation matrix in Table 3 shows a significant positive correlation between stress, anxiety, and depression, with a correlation coefficient of 0.88 between stress and anxiety, 0.86 between anxiety and depression, and 0.88 between stress and depression (*p* < 0.001), indicating a high degree of coexistence of these negative psychological symptoms among individuals. Among the inflammatory indicators related to HDL, MHR is positively correlated with stress, anxiety, and depression, with values of 0.23, 0.24, and 0.19, respectively (*p* < 0.001). The correlation coefficients between LHR and stress, anxiety, and depression are relatively low, at 0.11, 0.12, and 0.07, respectively (*p* < 0.001). The correlation between PHR and these negative psychological symptoms is also positively correlated, with values of 0.13, 0.16, and 0.11, respectively (*p* < 0.001). The correlation between NHR and stress, anxiety, and depression is also low, at 0.11, 0.11, and 0.08, respectively (*p* < 0.001). This suggests that the HDL-related inflammatory markers involved in this study are associated with anxiety, stress, and depression, offering dependable leads for subsequent analysis.

**Table 3:** The correlation matrix between negative psychological symptoms and HDL related inflammatory indicators.

	Stress	Anxiety	Depression	MHR	LHR	PHR	NHR
Stress	1.00						
Anxiety	0.88^***^	1.00					
Depression	0.88^***^	0.86^***^	1.00				
MHR	0.23^***^	0.24^***^	0.19^***^	1.00			
LHR	0.11^***^	0.12^***^	0.07^**^	0.69^***^	1.00		
PHR	0.13^***^	0.16^***^	0.11^***^	0.60^***^	0.68^***^	1.00	
NHR	0.11^***^	0.11^***^	0.08^**^	0.71^***^	0.66^***^	0.64 ^***^	1.00

***p < 0.001; **p < 0.01; *p < 0.05.

**Figure 2:**
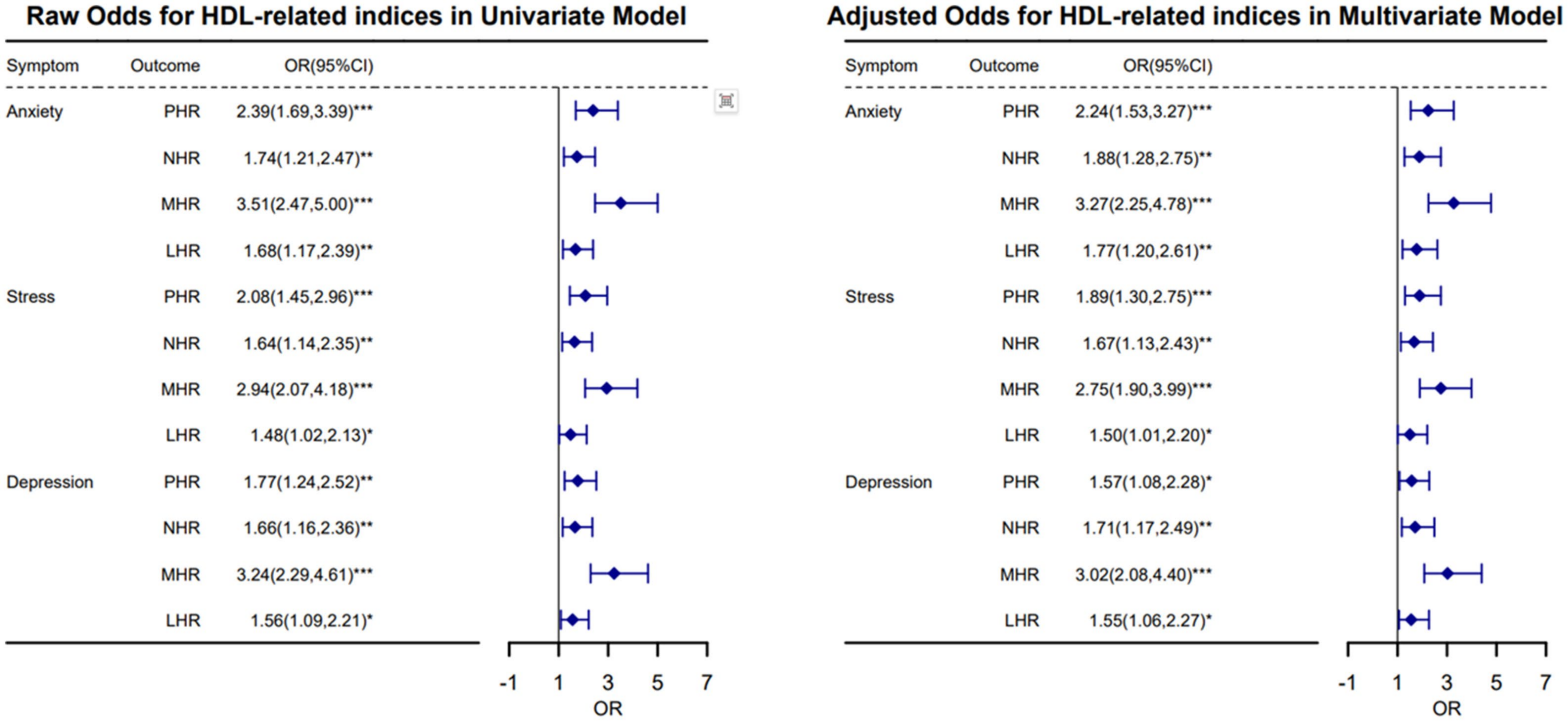
Forest plot of negative psychological symptoms in HDL-related inflammatory indicators.

Figure 2 presents the odds ratios and their associated 95% confidence intervals from both unadjusted (Model 1) and adjusted (Model 2) logistic regression analyses. These analyses explore the effect of negative psychological symptoms on HDL-related inflammatory indexes. Considered potential confounding factors such as gender, age, body mass index (BMI), education level, marital status, smoking, alcohol consumption, monthly income, night shift work, and working hours.

In the univariate model, the OR values for anxiety symptoms with PHR, NHR, MHR, and LHR were 2.39, 1.74, 3.51, and 1.68, respectively, all with statistical significance (*p* < 0.01). Stress symptoms were significantly associated with PHR, NHR, and MHR, with OR values of 2.08, 1.64, and 2.94 (*p* < 0.01), and a marginally significant association with LHR (OR = 1.48, *p* < 0.05). Depression symptoms showed significant associations with all four outcomes, with OR values of 1.77, 1.66, 3.24, and 1.56 (*p* < 0.05).

After adjusting for confounding factors, anxiety symptoms remained significantly associated with all four outcomes, with OR values of 2.24, 1.88, 3.27, and 1.77 (*p* < 0.01). Stress symptoms were significantly associated with PHR, NHR, and MHR (OR = 1.89, 1.67, 2.75, *p* < 0.01), and marginally significantly associated with LHR (OR = 1.50, *p* < 0.05). Depression symptoms continued to show significant associations with all four outcomes, with OR values of 1.57, 1.71, 3.02, and 1.55 (*p* < 0.05). The regression analysis findings indicate that individuals experiencing negative psychological states exhibited a moderate increase in the odds ratio for an abnormal high HDL-related inflammation index. This suggests a genuine association between these emotional symptoms and inflammatory conditions.

### Subgroup analysis

Given the unique and persistent psychological stressors faced by different occupational groups, we categorized these groups based on their job characteristics. This approach allowed us to examine the correlation between negative psychological symptoms and HDL-related inflammatory indices within each subgroup, aiming to pinpoint individuals at high risk of psychological - induced health damage.

Our subgroup analysis covered various factors such as work shift (night shift and working hours), monthly income, work type, and work age. For each subgroup, we constructed logistic regression models to evaluate the associations among the HDL-related inflammatory indices. By calculating and comparing the odds ratios across these subgroups, we gained insights into how different occupational factors influence the relationship between psychological symptoms and inflammatory indices. The specific results are outlined in Supplementary Figure S1.

The results show that anxiety, stress, and depression are risk factors for different subgroups of workers in MHR, and as monthly income decreases, work experience and working hours increase, workers with symptoms of anxiety, depression, and stress have an increased risk of abnormally elevated MHR, the risk of elevated MHR among technicians and medical staff with negative psychological symptoms is significantly higher than that of normal ones, with an OR value range of 1.70 ~ 2.65. To PHR, night work, income, and work hours emerge as significant determinants. Individuals in the low-income bracket (monthly earnings below 3,000 yuan) exhibited approximately twice the risk of abnormal elevation in PHR associated with anxiety and stress compared to the reference group. Employees who work night shifts 3–4 times weekly are at an increased risk of abnormal PHR elevation due to stress, depression, or anxiety, with odds ratios ranging from 2.79 to 5.63, compared to those in standard day-shift roles. For employees working more than 40 h per week, negative psychological symptoms significantly augmented the risk of elevated PHR levels. In terms of occupation, negative psychological symptoms among administrants were identified as risk factors for PHR abnormalities, with odds ratios ranging from 3.22 to 4.81.

For the risk of NHR, hazardous effects of anxiety and depression are predominantly manifest in populations subjected to night shifts and those with work years 20 ~ 30 years. Specifically, under conditions of anxiety, OR for NHR elevation increased by 0.64 for individuals engaged in night work 3 ~ 4 times/week compared to those 1 times/week. For the work year 20 ~ 30 years, both anxiety and depression were found to significantly elevate the risk of increased NHR levels, with ORs of 3.13 and 2.89, respectively (*p* < 0.05). Moreover, individuals experiencing mild overtime (working 41–48 h per week) with symptoms of anxiety and depression were at an elevated risk of NHR increment, with ORs of 2.21 and 2.50, respectively, (*p* < 0.05). In an intriguing observation, the phenomenon of elevated LHR appears to be associated with negative psychological symptoms particularly in younger individuals. Among workers whose work years ranging from 1 to 10, the risks of increased LHR due to anxiety, depression, and stress were 2.16, 1.87, and 1.75, respectively (*p* < 0.05). The elevation of LHR in response to anxiety and stress was also evident in individuals working night shifts 3 to 4 times per week, those with an income ranging from 5,001 to 10,000 yuan, and those employed in administrators. The increment in LHR associated with depression may be linked to income levels, as individuals with a monthly income between 3,001 ~ 5,000 yuan exhibited a 2.04-fold increased risk of abnormal LHR compared to their non-depressed counterparts.

### Receiver operating characteristic curve analysis

**Figure 3:**
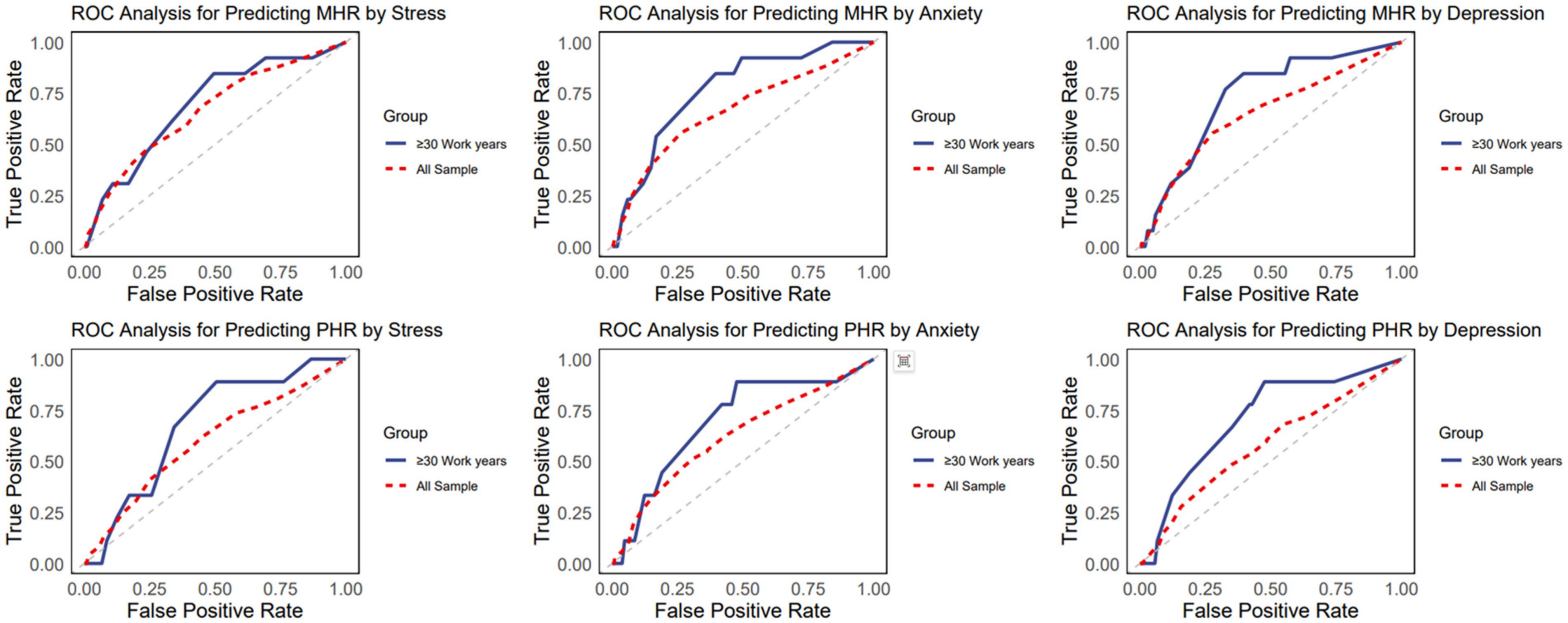
Receiver operating characteristic curve of negative psychological symptoms for HDL-related inflammatory indicators.

Table 4 shows the predictive ability indicators of anxiety, stress, and depression for HDL related inflammatory markers, with AUC greater than 0.6. All three psychological symptoms showed statistically significant associations (*p* < 0.01) with both MHR and PHR. Anxiety and stress exhibited similar predictive performance for MHR (AUC = 0.66), while depression also showed comparable predictive ability (AUC = 0.65). Anxiety’s predictive capacity for PHR was slightly lower (AUC = 0.61), and stress has shown its predictive for PHR whose AUC equals 0.60. These findings suggest a potential link between negative psychological states and HDL-related inflammation, warranting further investigation into the underlying mechanisms. Notably, the predictive performance AUC for those with over 30 years of work experience exceeds 0.7. Within this subgroup, anxiety and depression symptoms show the highest AUC for abnormally elevated MHR, at 0.77 and 0.73, respectively. This suggests that, for people with longer work tenure, monitoring their psychological state and internal inflammation levels is more effective. The ROC curves of three negative psychological symptoms on four indicators are shown in Figure 3.

**Table 4:** Performance metric for negative psychological symptom in predicting HDL-related inflammation indexes.

Symptom	Outcome	Cutoff	AUC	ACC	SEN	SPE	Youden index
Anxiety	MHR	13.5	0.66	0.72	0.56	0.74	0.29
Stress	MHR	15.5	0.66	0.74	0.47	0.77	0.24
Depression	MHR	13.5	0.65	0.71	0.56	0.72	0.28
Anxiety	PHR	11.5	0.61	0.66	0.53	0.67	0.20
Stress	PHR	11.0	0.60	0.55	0.62	0.55	0.17

## Discussion

This study investigated the relationship between HDL-related Inflammatory Indices and the prevalence of stress, anxiety, and depression among employees in different industries. Employing a cross-sectional design, the study collected data on participants’ demographic characteristics, work-related factors, and negative psychological symptoms through validated questionnaires, alongside blood samples for the analysis of HDL-related Inflammatory Indices. The findings revealed significant associations between the indices, particularly MHR and PHR, and the presence of stress, anxiety, and depression. Furthermore, preliminary analysis suggested that MHR may hold promise as a potential biomarker for predicting inflammation status due to these psychological symptoms, warranting further investigation into its utility in occupational mental health assessments.

The stress response involves the hypothalamic–pituitary–adrenal axis (HPA) and sympathetic nervous system, releasing hormones such as cortisol to alter lipid metabolism and promote inflammation (66–69). Previous studies have shown that chronic psychological stress response activates the HPA axis and the sympathetic nervous system to release hormones such as cortisol, thereby increasing the production of very low-density lipoprotein (VLDL) particles and reducing HDL-C (30, 70). Long term, chronic exposure to high cortisol may increase oxidative stress through various mechanisms, and chronic inflammation can alter the composition and function of HDL, leading to a decrease in HDL-C. Besides, HDL interacts with various immune cells, including macrophages, T cells, and B cells, to regulate their function (71, 72). When activated, immune cells such as macrophages and T lymphocytes release pro-inflammatory cytokines, disrupting lipid metabolism and exacerbating inflammation (73, 74). This immune response not only disrupts lipid homeostasis, but also enhances the activation of the HPA axis, thereby forming an inflammatory feedback loop.

HDL-C is primarily involved in lipid metabolism and is associated with conditions like cardiovascular disease. However, several factors limit its use as a broad health indicator. Firstly, the relationship between HDL-C and cardiovascular disease risk is not linear, but rather exhibits a “U”-shaped curve, where both high and low levels of HDL-C are associated with an increased risk of all-cause mortality (75). Secondly, the concentration of HDL-C can be influenced by dietary structure, exercise habits, and the body’s oxidative stress status (76–78), which may result in low specificity when used as a systemic health indicator. The interaction between HDL-C levels and other health risk factors also plays a crucial role. For instance, an individual with high HDL-C levels may not experience a reduced disease risk if other significant cardiovascular risk factors are present, such as high platelet concentration, endothelial damage, and increased monocyte migration (79–83). Thus, considering HDL-C levels alone while ignoring the combined effects of other risk factors can result in an inaccurate evaluation of individual cardiovascular risk.

Persistent exposure to negative psychological states can influence the physiological mechanisms of the human body, with notable impacts on the immune and hematological systems (84). Enduring stress elicits a response from the innate immune system, precipitating the migration of peripheral myeloid cells (chiefly monocytes and neutrophils) and the synthesis of pro-inflammatory cytokines, such as interleukin-6 (IL-6), which subsequently ignites an inflammatory cascade (85). Empirical evidence indicates that individuals suffering from depression present with an augmented monocyte count in peripheral circulation, alongside an altered neutrophil-to-lymphocyte ratio (86). These inflammatory responses not only compromise immune homeostasis but may also permeate the blood–brain barrier, thereby intensifying neuropsychiatric disorders like depression and anxiety. The rise in peripheral blood mononuclear cells and platelets in response to chronic and socio-psychological stress is intricately associated with inflammatory and neuroimmune pathways, implying that immune system activation could be a critical intermediary in the complex interplay between stress, anxiety, and depression (87–89).

NHR reflects the interrelationship between neutrophil counts and HDL levels. Neutrophils are a critical cell type in the immune system, involved in inflammatory responses and defense against infections (90). The MHR quantifies the association between monocyte counts and HDL levels. Given the pivotal role of monocytes in the pathogenesis and progression of atherosclerosis, their dysregulation in quantity and function may contribute to cardiovascular diseases. LHR elucidates the correlation between lymphocyte counts and HDL levels, with lymphocytes playing a significant role in modulating immune responses and maintaining homeostasis. PHR represents the relationship between platelet counts and HDL levels, where platelets are essential in thrombosis and inflammatory reactions, while HDL exerts anti-inflammatory and antithrombotic properties. These four indices have been extensively explored and their applicability has been validated in the context of cardiovascular diseases and metabolic syndrome (91, 92).

These additional findings reinforce the concept that HDL related inflammatory markers may be biomarkers for negative psychological effects of work-related issues. Inflammatory mediators such as thrombopoietin, interleukin, and TNF-α can regulate the production and release of platelets and white blood cells in the blood, which may lead to changes in these cell counts (93–95). During the acute phase of inflammation, activated white blood cells can promote the inflammatory process by releasing substances that exacerbate inflammation. The changes in blood cell count mainly serve as non-specific markers of inflammation, but the combination of a series of inflammatory indicators such as HDL-C can expand our understanding of the complex relationship between blood cells, lipid metabolism, and inflammatory response, further linking systemic inflammation with the physiological manifestations of negative psychological syndrome.

Our study showed that MHR and PHR has the strongest correlation with stress, anxiety, and depression. This aligns with the research conducted by Marsland, A. L., who discovered that stress and negative emotions can lead to an elevation in cardiovascular inflammatory markers (96). Furthermore, this finding resonates, to a certain degree, with our own results indicating that MHR and PHR can serve as indicators of stress, as well as states of anxiety and depression. However, the association between HDL and negative psychological symptoms, such as occupational stress and burnout, has been well-documented in numerous studies (32, 39, 45, 47). Specifically, chronic occupational psychological stress is implicated in metabolic dysregulation and enhances the susceptibility to cardiovascular disease. MHR and PHR showed significant correlations with stress, anxiety, and depression in different subgroups, this suggests that the burden of these psychological states may be reflected by inflammatory markers in most employees. In contrast to previous studies, the research by Wei et al. reported an elevation of the MHR during manic episodes in bipolar disorder, while it was found to be the lowest in the bipolar depression group. Remarkably, the elevation of the PHR within the bipolar depression group corresponds with the observations of the current investigation (55). Nonetheless, the study by Wei et al. focused exclusively on individuals with schizophrenia and bipolar disorder, without incorporating a healthy control group. Consequently, a direct comparative analysis with the present research is precluded, and the results must be considered as reference rather than directly comparable phenomena.

Interestingly, in the subgroup analysis of this study, the risk of MHR abnormally increasing due to adverse psychological symptoms also increased with the increase of work experience, and the risk of NHR significantly increased in the anxiety and depression group compared to the normal group in the 20–30 years work experience group. This finding suggests that MHR and NHR have higher practical significance in predicting negative psychological symptoms in older adult employees, consistent with previous research where MHR has demonstrated superior predictive efficacy in middle-aged and older populations for other conditions, such as cardiovascular and cerebrovascular diseases, as well as metabolic syndrome (91, 97). The association between NHR and depression has been more extensively studied compared to other indices. Similar to the findings of Li et al., this study reveals that the correlation between NHR and depressive status, as well as its predictive power, intensifies with advancing age (98). In Chinese cohort studies, NHR also demonstrated a stronger association and higher predictive efficacy for the development of type 2 diabetes mellitus in the middle-aged and older group (99, 100).

In addition, night shifts and long working hours are also important factors affecting the HDL-related Inflammatory Indices. In all four different indicators, there is a risk of increased inflammation index when negative psychological symptoms occur in the working population with night shifts and overtime. However, work hours did not show a complete positive correlation in the dose–response relationship like work years, and there was a U-shaped curve relationship, which is consistent with previous research. Niu et al. found that mental disorders and working hours did not show a completely linear relationship (12), but rather a complex nonlinear relationship, related to age and gender. It is suggested that more comprehensive health monitoring and intervention measures should be carried out for people with poor work systems. Similar to previous studies, income is also an important grouping factor affecting inflammation status. Low-income groups are more likely to experience various psychological symptoms such as anxiety, depression, and stress, and the risk of elevated inflammatory markers is also higher than other groups (101). In this study, MHR, PHR, and LHR all showed this phenomenon. The health risks of low-income groups have always been a focus of attention for health institutions, which need to take comprehensive measures from multiple perspectives such as economy, psychology, and chronic disease management to reduce these risks and improve the overall health level of low-income groups.

Furthermore, our study found that PHR and MHR, as potential biomarkers, have certain specificity in monitoring mental health problems in occupational populations. They are closely related to occupational stressors and reflect the potential connection between physical inflammation and psychological stress. Different from other general biomarkers, they can be used as prospective monitoring indicators for occupational health in the future. A study compared biomarkers such as MHR and PHR with inflammatory indicators such as interleukin-25 (49), and the results showed a good correlation between the two, which can reflect the inflammatory state in the human body (51). However, compared to interleukin, HDL related inflammatory indicators are indeed more economical, convenient, and easier to promote. Consistent with previous studies, within older subgroups, the predictive power and correlation between psychological symptoms and inflammatory indicators are more significant. In our study, the AUC of the group with work experience greater than 30 years old increased by about 10% compared to the entire sample. This also indicates that in the aging society, HDL related indicators can be included in the health monitoring of the older adult working population to detect their health damage early and intervene to prevent the occurrence and development of diseases.

## Advantages and limitations

The advantages of our research are listed as follows: (1) Our study covers multiple occupational groups such as teachers, medical staff, administrators and workers, with a moderate sample size. After adjusting for confounders, the research results remain robust, indicating that our findings have a certain degree of stability and universal applicability. (2) The study conducted in-depth analysis on specific subgroups and further explored the applicability of relevant indicators. (3) The indicators we have included are high cost-effective and can be followed up for a long time for the professional population, which has certain reference significance for future research. (4) Compared to traditional questionnaires, the biological indicators in this study are more objective and feasible for companies to collect and monitor. By tracking employees’ mental health and HDL—related inflammatory markers, companies can accurately grasp health trends and the risk of chronic diseases like cardiovascular disease. This enables them to better safeguard employees’ health.

At the meanwhile, the limitations of the research seem noticeable, (1) as a cross-sectional investigation, the findings presented herein should be interpreted as associative rather than causal with respect to the relationship between the examined indicators and negative psychological symptoms, such as tension, anxiety, and depression. To determine if psychological symptoms are indeed responsible for the abnormal increase in HDL-inflammatory factors, it is advisable to conduct either a cohort study or a clinical intervention research design. (2) From the perspective of biopsychosocial models, the interplay between inflammation and psychological symptoms is likely to be characterized by bidirectional causality, necessitating additional studies to elucidate the underlying mechanisms. While the associations found were statistically significant, the predictive ability of the biomarkers remains moderate (AUC > 0.6). These biomarkers are not yet sufficient as standalone clinical diagnostic tools. As biomarkers for negative psychological symptoms, the identified indicators could be further validated through comparison with more comprehensive clinical measures, including brain imaging studies and cerebrospinal fluid protein analysis. (3) In the present study, stress, anxiety, and depression were operationalized using self-report scales, thus the outcomes are conceptualized as negative psychological symptoms. Should these be classified as stress disorders, anxiety disorders, or major depressive disorders, diagnosis by qualified clinical professionals would be required. Future research will be directed toward a more nuanced exploration informed by clinical diagnoses. (4) This investigation considered individual attributes and job-related characteristics as confounding variables and adjusted for their influence. Nevertheless, constrained by the nature of cross-sectional survey methodology, we encountered challenges in precisely capturing the historical mental health aspects of the participants, including social support networks, psychological capital, and prior mental health backgrounds. As a result, our analysis was unable to provide a complete explanation of the interplay between psychological state and the subsequent physiological strain. New studies would benefit from integrating a more extensive array of psychological data to enhance understanding.

## Conclusion

This study demonstrates a significant correlation between HDL-related Inflammatory Indices, particularly MHR and PHR, and the prevalence of stress, anxiety, and depression among healthcare professionals. The findings suggest that MHR and PHR may serve as a potential biomarker for identifying individuals at risk of developing these psychological symptoms. Therefore, incorporating these indices into occupational mental health assessments could provide valuable insights for early intervention and prevention strategies to mitigate the impact of stress, anxiety, and depression in the healthcare sector. Further research is needed to validate these preliminary findings and explore the underlying mechanisms linking inflammation to mental health outcomes.

## Data Availability

The raw data supporting the conclusions of this article will be made available by the authors, without undue reservation.
