# Supplementary figures and images for: Monitoring of mental health in occupational populations: a study on the role and application of HDL-related inflammatory index
Source: Front Public Health. 2025 Mar 31;13:1563742. doi: 10.3389/fpubh.2025.1563742 (PMC11994679; doi:10.3389/fpubh.2025.1563742)

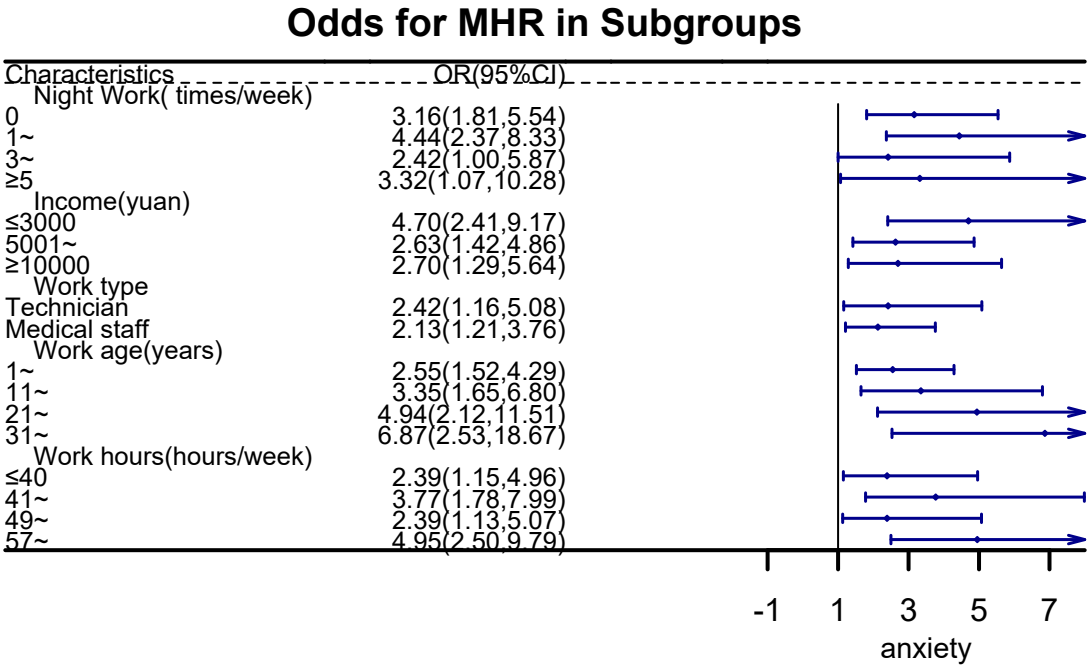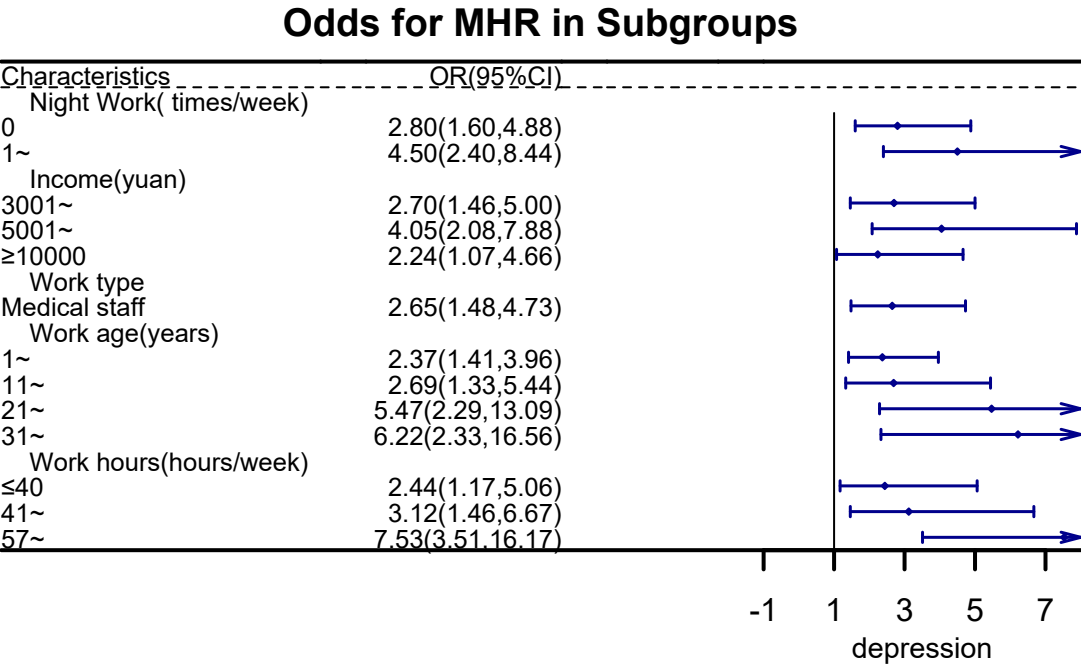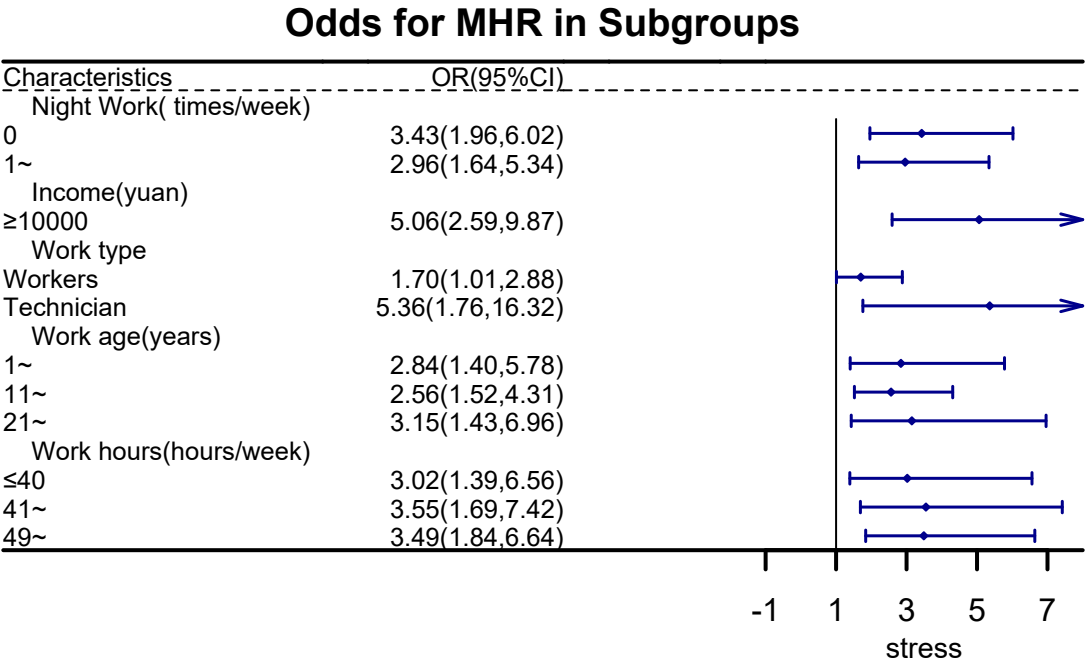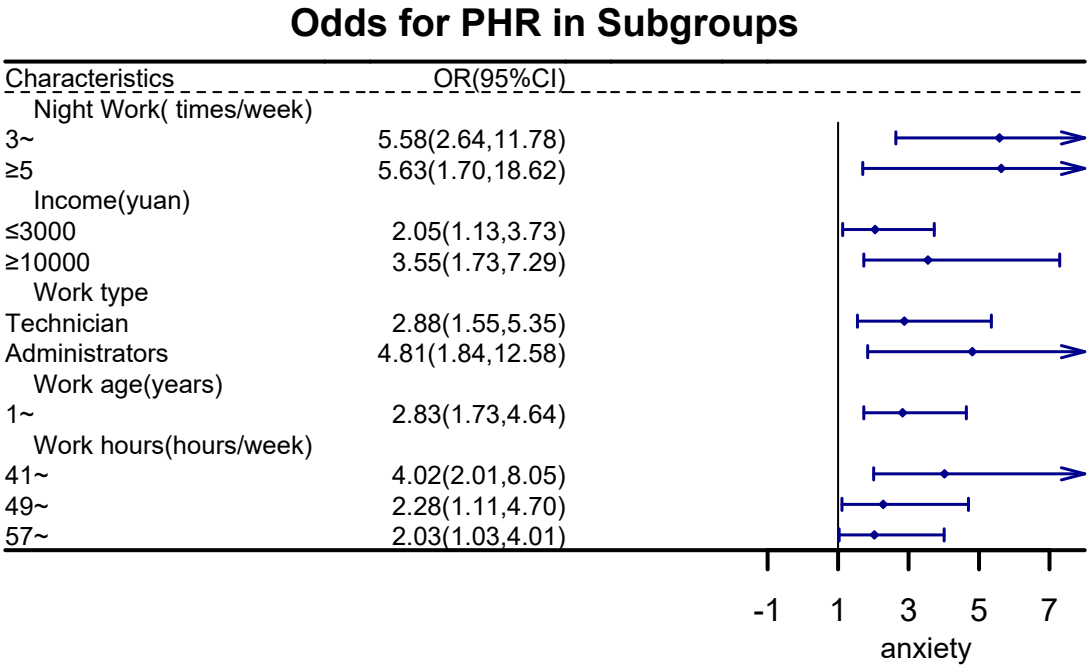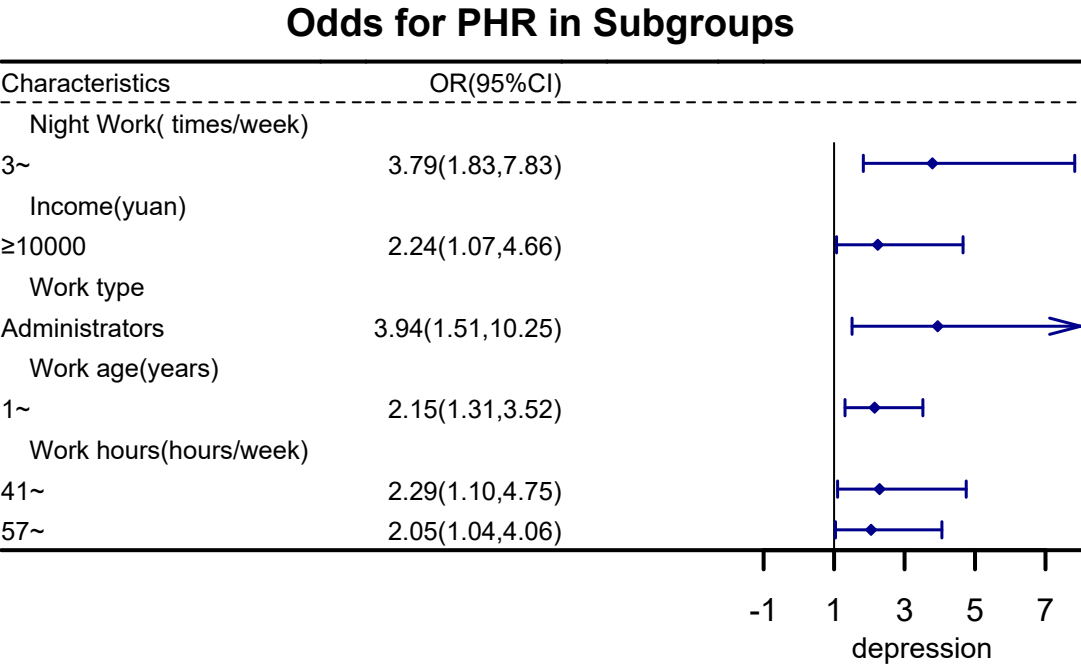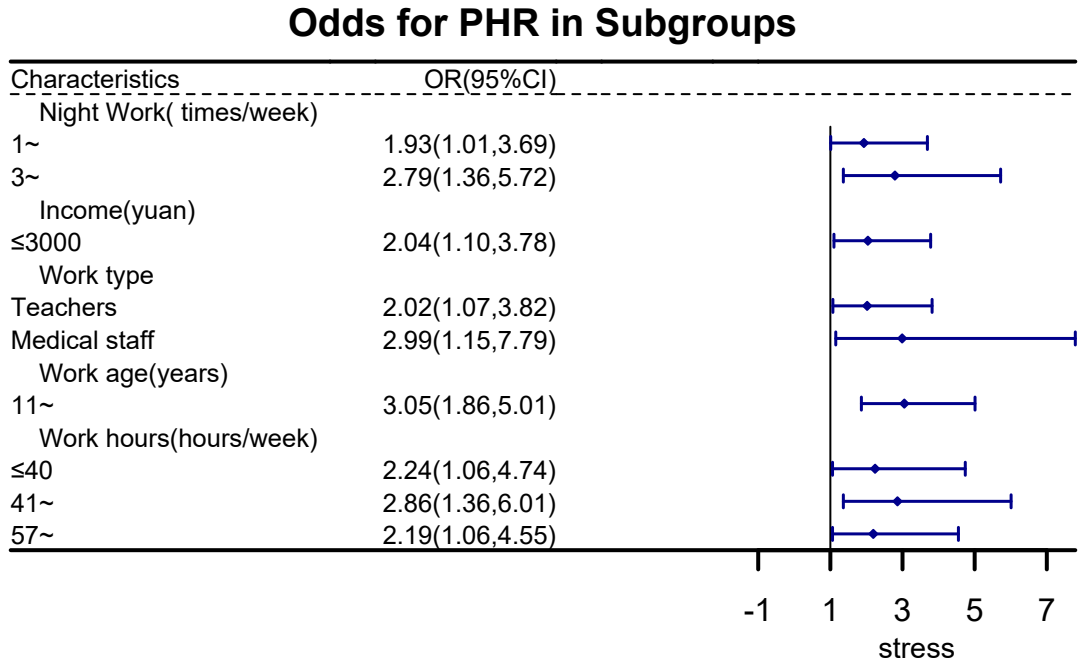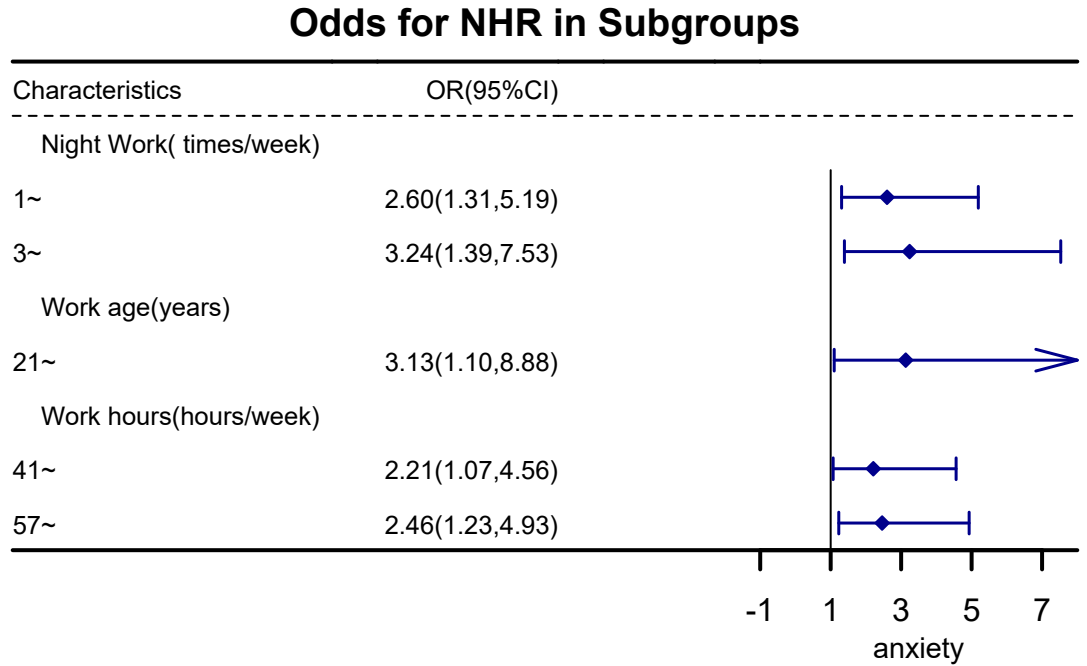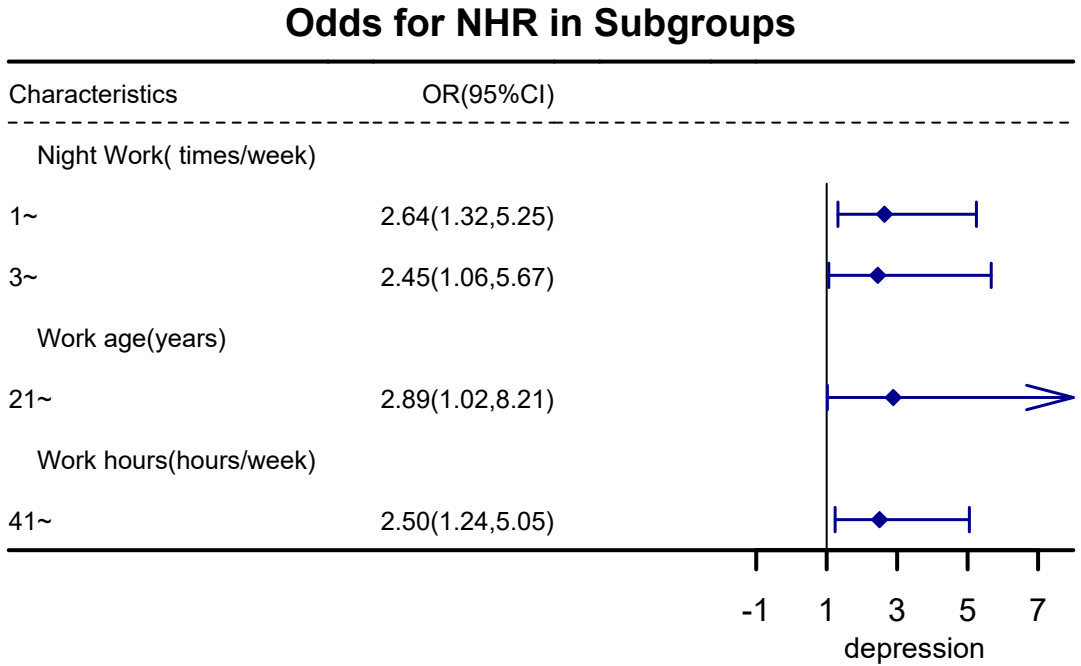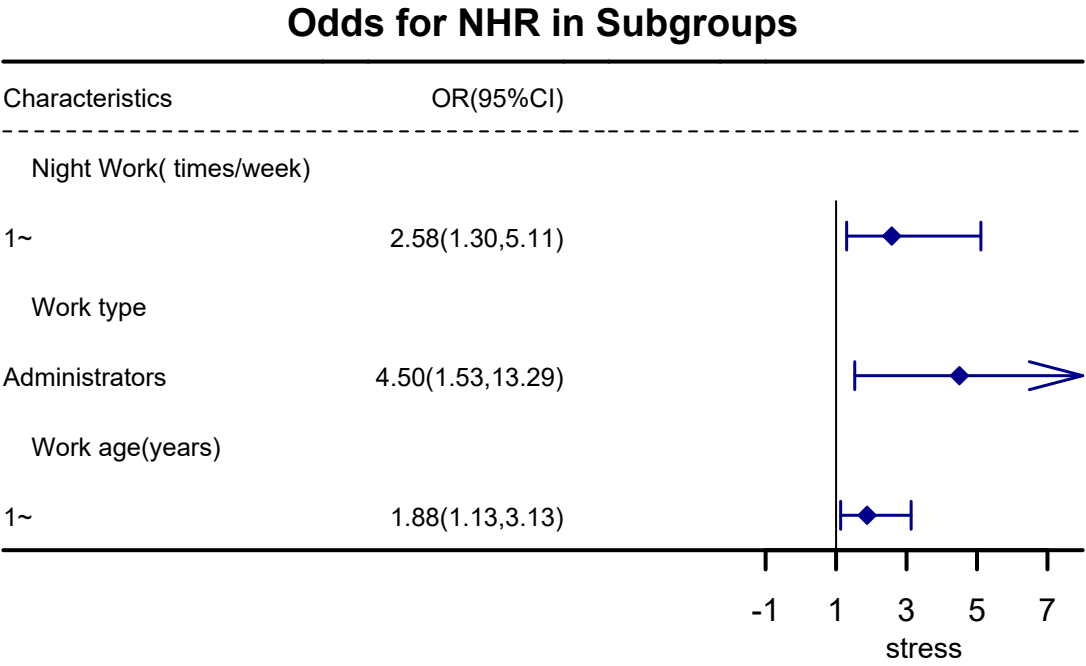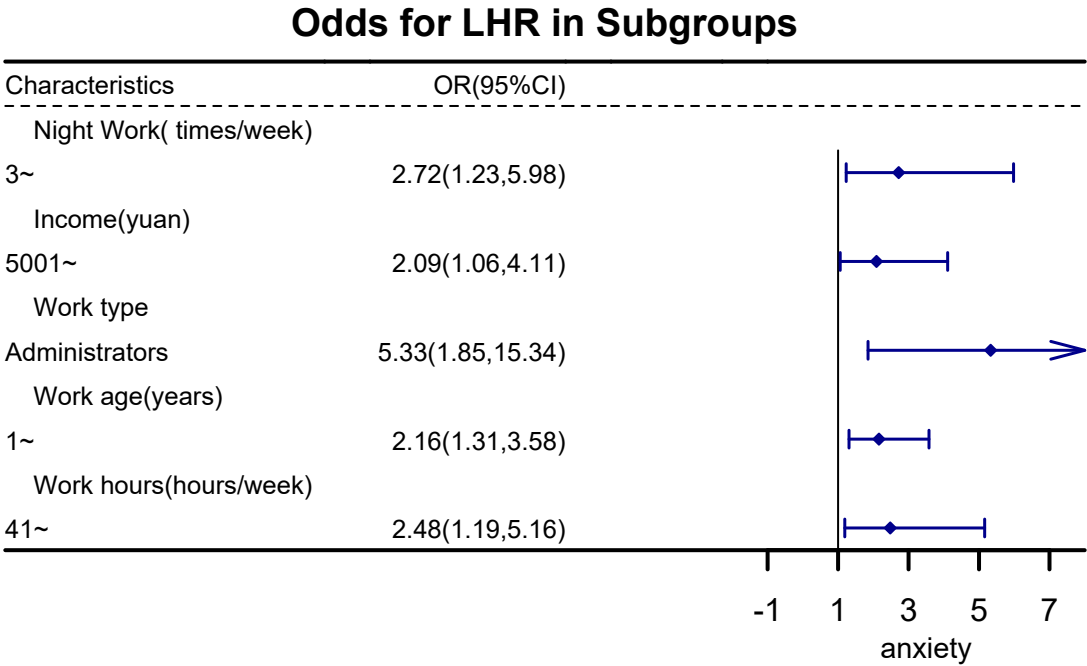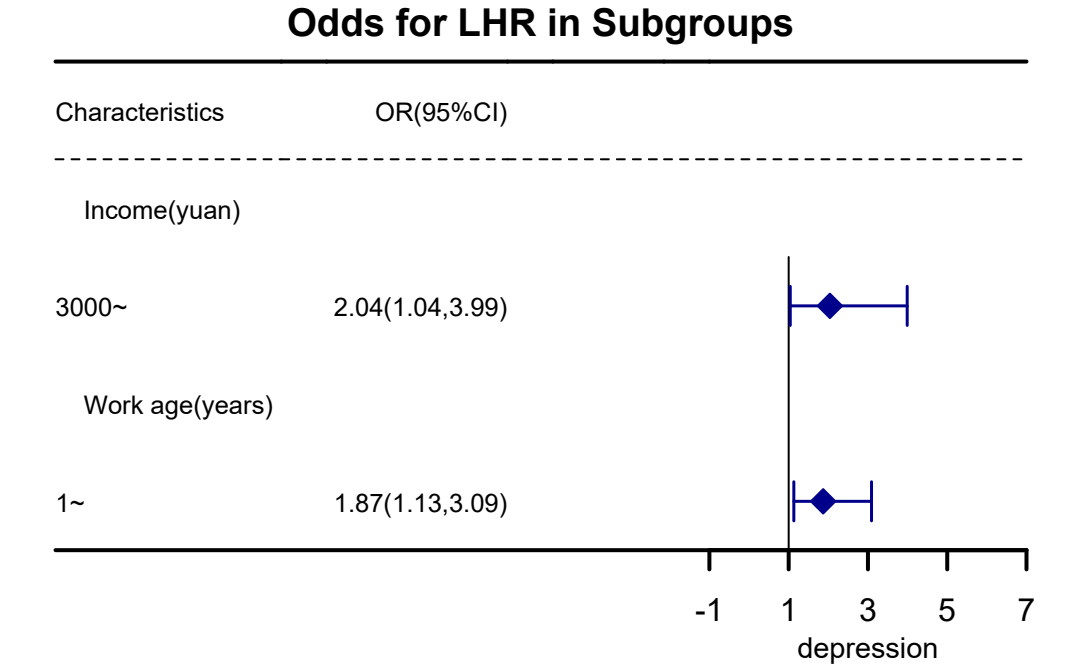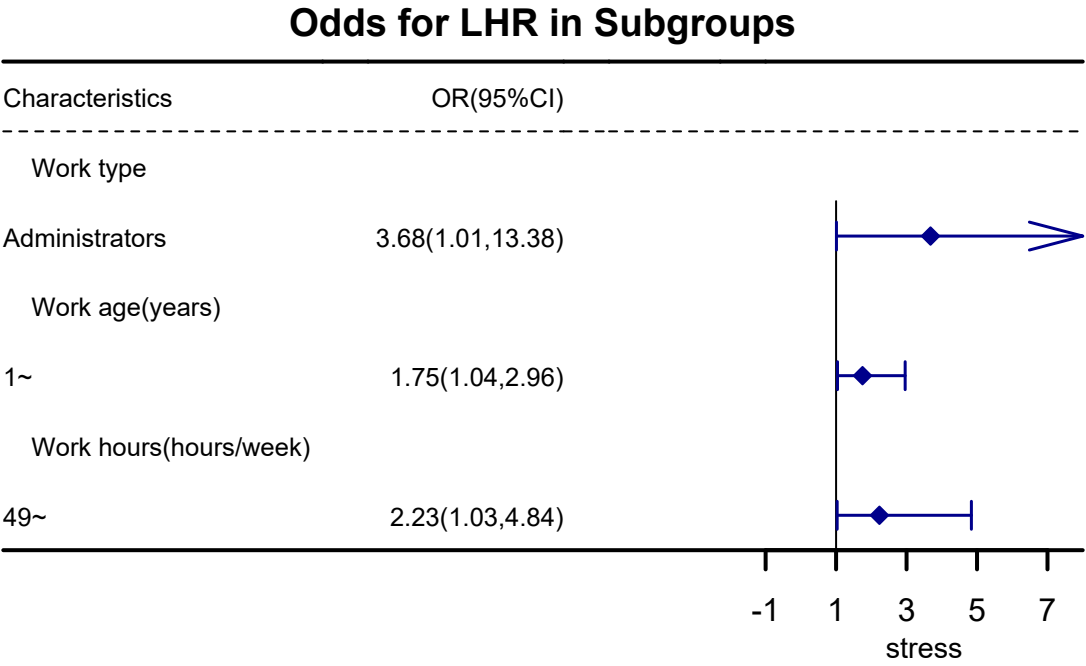

Supplement: Supplementary file 1 [file Image_1.pdf]
