# Supplementary material for: Monitoring of mental health in occupational populations: a study on the role and application of HDL-related inflammatory index
Source: Front Public Health. 2025 Mar 31;13:1563742. doi: 10.3389/fpubh.2025.1563742 (PMC11994679; doi:10.3389/fpubh.2025.1563742)
Supplement: Supplementary file 2 [file Table_1.docx]

| Symptom | | PHR | NHR | MHR | LHR |
| --- | --- | --- | --- | --- | --- |
| Stress |  |  |  |  |  |
|  | No | 181.43±59.06 | 2.82±1.17 | 0.29±0.12 | 1.59±0.63 |
|  | Yes | 198.39±70.23 | 3.05±1.30 | 0.33±0.15 | 1.72±0.69 |
|  | t-test | 112.255 | 77.246 | 2.822 | 65.765 |
|  | P-Value | <0.001 | 0.002 | <0.001 | 0.001 |
| Anxiety |  |  |  |  |  |
|  | No | 180.51±58.41 | 2.83±1.15 | 0.28±0.12 | 1.58±0.62 |
|  | Yes | 198.83±70.12 | 3.01±1.31 | 0.33±0.15 | 1.72±0.71 |
|  | t-test | 112.236 | 76.028 | 0.285 | 63.58 |
|  | P-Value | <0.001 | 0.008 | <0.001 | <0.001 |
| Depression |  |  |  |  |  |
|  | No | 181.71±60.40 | 2.84±1.16 | 0.28±0.12 | 1.59±0.62 |
|  | Yes | 194.98±66.36 | 2.98±1.30 | 0.33±0.14 | 1.69±0.69 |
|  | t-test | 112.223 | 75.203 | 1.415 | 62.129 |
|  | P-Value | <0.001 | 0.036 | <0.001 | 0.009 |

Appendix Table 1 Comparation of HDL-related Inflammatory Indicators for Negative psychological symptoms
